# Effects of Thrombin-Based Hemostatic Agent in Total Knee Arthroplasty: Meta-Analysis

**DOI:** 10.3390/jcm12206656

**Published:** 2023-10-20

**Authors:** Jung-Wee Park, Tae Woo Kim, Chong Bum Chang, Minji Han, Jong Jin Go, Byung Kyu Park, Woo-Lam Jo, Young-Kyun Lee

**Affiliations:** 1 Department of Orthopaedic Surgery, Seoul National University Bundang Hospital, Seongnam 13620, Republic of Korea; jwepark@gmail.com (J.-W.P.); ccbknee@gmail.com (C.B.C.); gjjjl@naver.com (J.J.G.); hellowbk@gmail.com (B.K.P.); 2 Department of Orthopaedic Surgery, Seoul National University College of Medicine, Seoul 03080, Republic of Korea; orthopassion@naver.com; 3 Department of Orthopaedic Surgery, SMG-SNU Boramae Medical Center, Seoul 07061, Republic of Korea; 4 Department of Health Science and Technology, Graduate School of Convergence Science and Technology, Seoul National University, Seoul 08826, Republic of Korea; mj830@snu.ac.kr; 5 Department of Orthopaedic Surgery, Seoul St. Mary’s Hospital, College of Medicine, The Catholic University of Korea, Seoul 06591, Republic of Korea

**Keywords:** total knee arthroplasty, thrombin-based hemostatic agent, hemostatic matrix, blood loss, transfusion, meta-analysis

## Abstract

The effectiveness of Floseal, a thrombin-based hemostatic matrix, in total knee arthroplasty (TKA) in minimizing blood loss and transfusion requirements remains a topic of debate. This meta-analysis aims to evaluate the up-to-date randomized controlled trials (RCTs) on the efficacy and safety of Floseal in TKA. A comprehensive search was conducted in electronic databases to identify relevant RCTs. The methodological quality of the included studies was assessed, and data extraction was performed. The pooled effect sizes were calculated using standardized mean difference (SMD) or odds ratios (OR) with 95% confidence intervals (CIs). Eight studies involving 904 patients were included in the meta-analysis. The use of a thrombin-based hemostatic agent significantly reduced hemoglobin decline (SMD = −0.49, 95% CI: −0.92 to −0.07) and the risk of allogenic transfusion (OR = 0.45, 95% CI: 0.25 to 0.81) but showed no significant difference in the volume of drainage or total blood loss. Funnel plots showed no evidence of publication bias. This meta-analysis provides robust evidence supporting the effectiveness of Floseal in reducing hemoglobin decline and transfusion in TKA. Further well-designed RCTs with longer follow-up periods are warranted to assess long-term efficacy and safety.

## 1. Introduction

Total knee arthroplasty (TKA) is a satisfactory surgical option in elderly patients with intractable symptoms and advanced osteoarthritis of the knee joint [1,2]. However, TKA is usually associated with significant perioperative blood loss and an increased need for allogenic blood transfusion because it requires soft tissue exposure, extensive bony resection, and a lengthy operation time [3,4]. The acute anemia and the allogeneic blood transfusion used to treat the anemia could lead to perioperative comorbidities and increase medical costs [5,6]. Therefore, surgeons prioritize minimizing perioperative blood loss and have employed various methods to achieve this goal and reduce the need for blood transfusion following TKA. These methods include the use of erythropoietic agents, autologous blood transfusion from pre-donated blood, cell salvage, hemostatic agents, and antifibrinolytic agents [7]. 

One of the widely used methods is the administration of Floseal (Baxter, Deerfield, IL, USA), a hemostatic matrix composed of thrombin and bovine gelatin, which can promote blood coagulation [8]. This thrombin-saturated gelatin plays a role in the initial hemostasis process where the vessel injury due to surgery occurs. Aggregation of platelets and activating the coagulation pathway leads to the conversion of prothrombin to thrombin and the subsequent formation of insoluble fibrin composites [9]. When the thrombin-rich gelatin is applied to the bleeding site, it affects the coagulation process by not only creating a fibrin clot, activating and inducing the platelet aggregation but also triggering a tamponade effect that mechanically stops the bleeding by swelling the gelatin granules by 10% to 20% [10]. The function of gelatin in this composite is the excellent absorbent feature, which enables it to absorb and carry 200% of its volume in liquid [11]; this enhances the coagulation process by maximizing the local platelet concentration in the bleeding site and the efficient release of prothrombin kinase that is required in the coagulation cascade [12]. Thrombin-based hemostatic agents have been traditionally adopted in various fields, including general, cardiac, gynecologic, neurovascular, and orthopedic surgeries [8,13,14,15,16], and now are expanding their indication to otorhinolaryngologic, dental, and urologic surgeries [17,18,19,20,21]. 

In the scope of TKA, some clinical studies have shown the prominent effect of Floseal in terms of a decrease in perioperative bleeding or hemoglobin drop [22,23,24,25,26,27,28]. However, these studies suffer from methodological flaws, such as poor study design, small sample sizes, and inconsistent outcomes. Due to these flaws, the use of thrombin-based hemostatic agents in TKA is still a topic of debate, and there is a need for more reliable and convincing data to assess its efficacy and safety. There are two previous meta-analyses that incorporated the outcomes of thrombin-based hemostatic matrix use in TKA [29,30]. However, these studies were published in 2014 and 2017, and only four randomized clinical trials (RCTs) were included. Therefore, the aim of this study was to evaluate up-to-date RCTs on the effectiveness and safety of thrombin-based hemostatic agents in TKA.

## 2. Materials and Methods

### 2.1. Search Strategy

**Figure 1:**
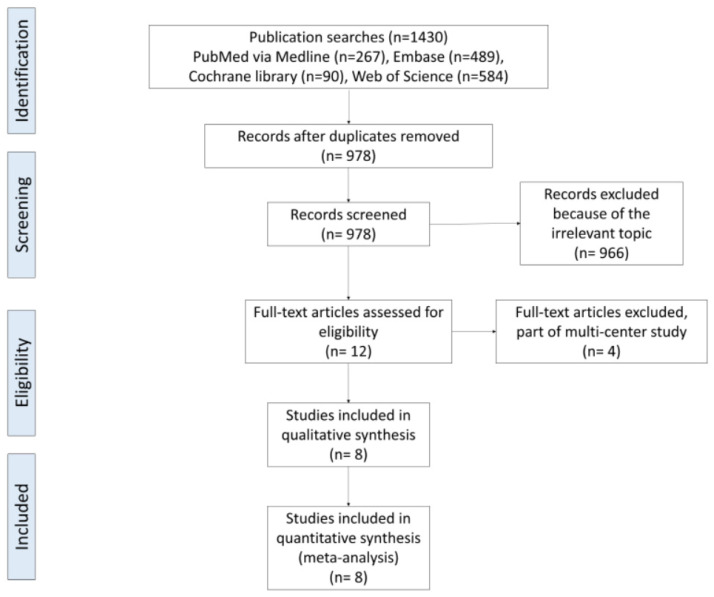
PRISMA flowchart of the systematic search. PRISMA, Preferred Reporting Items for Systematic Reviews and Meta-Analyses.

This study was conducted following the PRISMA (Preferred Reporting Items for Systematic Reviews and Meta-Analyses) guidelines but not registered in the International Prospective Register of Systematic Reviews (PROSPERO). Electronic databases, including PubMed, Embase Cochrane Library, and Web of Science, were searched. The systematic search was carried out in January 2023. There was no restriction on the publication date or the language. The search process was conducted as illustrated in Figure 1. 

Search terms were generated using the Boolean operators (AND or OR) and the keywords “thrombin” OR “Floseal” OR “hemostatic matrix” and “knee replacement” in combination. The search process was conducted by two reviewers separately, and in case of any disagreement, a third reviewer was consulted. To assess the methodological quality of the included literature, the risk of bias outlined in the Cochrane Handbook for Systematic Reviews of Interventions version 6.3 was used [31]. 

### 2.2. Selection Criteria

The inclusion criteria were as follows: (1) the studies on patients who received TKA; (2) the studies that used Floseal with comparison to the control group (control groups could be treated with other intervention or no intervention); (3) the studies that included outcomes relevant to patient blood management; and (4) the studies that were published RCTs. The studies were excluded if hemostatic agents other than Floseal were used in the experimental group. 

### 2.3. Data Extraction

The data extraction process was conducted independently by two researchers. They extracted various types of data from the included literature, such as the name of the first author, publication year, details of the interventions, demographics, number of included patients, and outcome measures. Additionally, other relevant parameters from individual studies were also extracted.

### 2.4. Data Analysis and Statistical Methods

Effect sizes were calculated based on the type of data: the standardized mean difference (SMD) was used for continuous data, calculated by dividing the mean difference (MD) by the common standard deviation (*SD*). For binary data, odds ratios (OR) were used. The pooled standard deviation (*SD*) was calculated by applying the following formula $\;SD=\sqrt{\frac{\left(n_{1}-1\right)\times {s_{1}}^{2}+\left(n_{2}-1\right)\times {s_{2}}^{2}}{\left(n_{1}+n_{2}-2\right)}}$, where *n*_1_ and *n*_2_ represent the sample sizes of the treatment and control groups, respectively, and *s*_1_ and *s*_2_ denote the standard deviations of the mean difference before and after treatment in the treatment and control groups, respectively [32].

Heterogeneity was estimated depending on the value of p and I^2^ using the standard chi-square test. When I^2^ > 50%, *p* < 0.1 was considered to be significant heterogeneity [33]. Therefore, a random-effect model was applied for data analysis. A fixed-effect model was used when no significant heterogeneity was found. To evaluate biases related to publication, we utilized funnel plots, which visually depict the characteristics and results of individual studies. We conducted a meta-analysis using Excel, a Microsoft application, and R (version 4.2.2). The data pooling process was performed in Excel, while the meta-analysis was conducted in R using the ‘meta’ and ‘metafor’ packages.

## 3. Results

### 3.1. Literature Search

A total of 1430 potential studies were identified with the first search strategy. Additionally, 452 duplicated articles were deleted, leaving 978 records. After screening, in total, 12 full-text articles were assessed for eligibility. Out of twelve, four reports were excluded according to the eligibility criteria. No additional studies were obtained after the reference review. Finally, eight independent comparison studies were eligible for data extraction and meta-analysis, as indicated by the flowchart in Figure 1 [22,24,25,26,27,28,34,35]. These studies involved a total of 485 patients in the Floseal group and 418 patients in the control group. 

### 3.2. Study Characteristics

The main characteristics of the included studies are reported in Table 1. All the studies evaluated primary TKA. Statistically similar baseline characteristics were observed between the Floseal and control groups, including age, sex, body mass index, preoperative hemoglobin, comorbidities, and anesthesia. In each study, thrombin-based hemostatic matrix was administered intra-articularly before suturing, though the dosages varied (5–10 mL). 

### 3.3. Risk of Bias Assessment

The included trials had small sample sizes, ranging from 10 to 157 patients; however, they were relatively well-designed and well-implemented. The quality of the included studies, according to the Cochrane Handbook for Systematic Reviews of Interventions, is reported in Table 2.

In four studies [24,27,28,35], random numbers generated by a computer and proper concealment of allocation were used, and two studies [27,28] implemented a double-blind approach involving blinding of participants and personnel.

All the included studies did not have an unclear bias due to incomplete outcome data or selective outcome reporting.

### 3.4. Outcomes for Meta-Analysis

#### 3.4.1. Hemoglobin Decline

**Figure 2:**
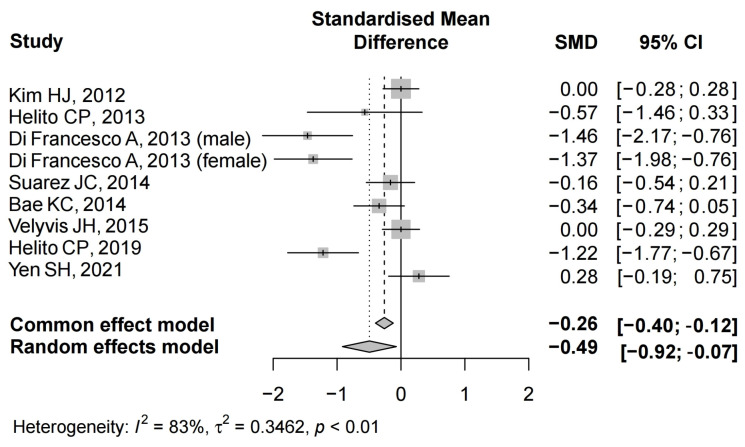
The forest plot of hemoglobin decline from the included studies [22,24,25,26,27,28,34,35].

Details regarding hemoglobin decline after TKA were available in all eight studies [22,24,25,26,27,28,34,35]. Two studies demonstrated a significant difference between the groups [24,25]. There was significant heterogeneity (I^2^ = 83%, *p* < 0.01); therefore, a random-effect model was performed. The pooled results showed that hemoglobin decline in the Floseal group was significantly lower than that in the control group (SMD = −0.49, 95% CI: −0.92 to −0.07) (Figure 2).

#### 3.4.2. Volume of Drainage

**Figure 3:**
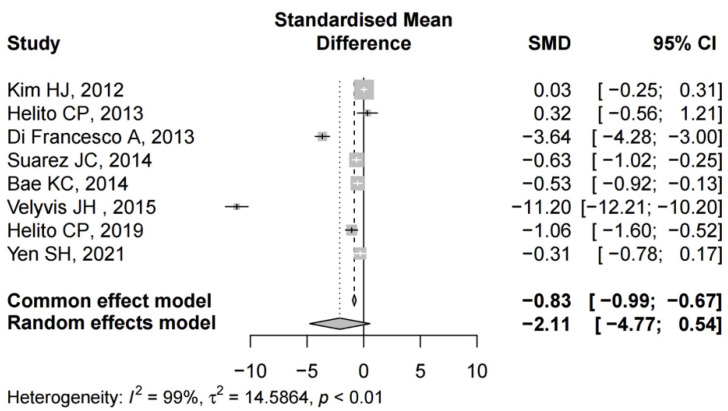
The forest plot of the volume of drainage from the included studies [22,24,25,26,27,28,34,35].

Details regarding the volume of drainage after TKA were available in all studies [22,24,25,26,27,28,34,35]. Five studies demonstrated a significant difference between the groups [22,24,25,28,34]. There was significant heterogeneity (I^2^ = 99%, *p* < 0.01); therefore, a random effect model was performed. The pooled results showed that there was no significant difference in drainage between the two groups (SMD = −2.11, 95% CI: −4.77 to 0.54) (Figure 3).

#### 3.4.3. Total Blood Loss

**Figure 4:**
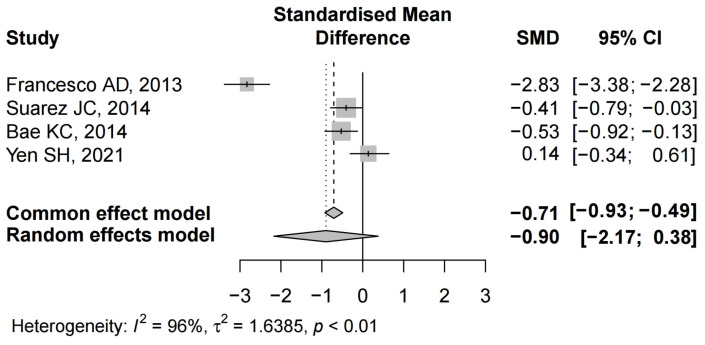
The forest plot of total blood loss from the included studies [22,24,25,28,35].

Details regarding total blood loss after TKA were available in five studies [22,24,25,28,35]. Three studies demonstrated a significant difference between the groups [22,24,28]. There was significant heterogeneity (I^2^ = 96%, *p* < 0.01); therefore, a random-effect model was performed. There was no significant difference in total blood loss between the two groups (SMD = −0.90, 95% CI: −2.17 to 0.38) (Figure 4).

#### 3.4.4. Risk of Allogenic Transfusion

**Figure 5:**
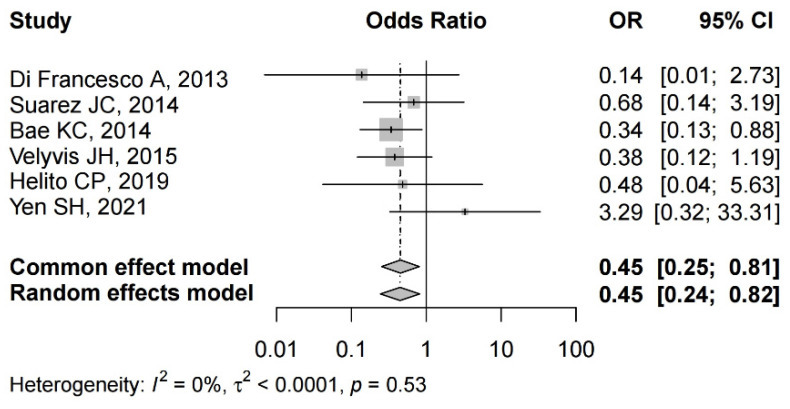
The forest plot of allogenic blood transfusion from the included studies [22,24,25,28,34,35].

Details regarding transfusion rate after TKA were available in six studies [22,24,25,28,34,35]. One study demonstrated a significant difference between the groups [22]. There was no significant heterogeneity (I^2^ = 0%, *p* = 0.53); therefore, a common effect model was performed. The pooled results showed that the transfusion rate in the Floseal group was significantly lower than that in the control group (OR = 0.45, 95% CI: 0.25 to 0.81) (Figure 5).

### 3.5. Publication Bias

**Figure 6:**
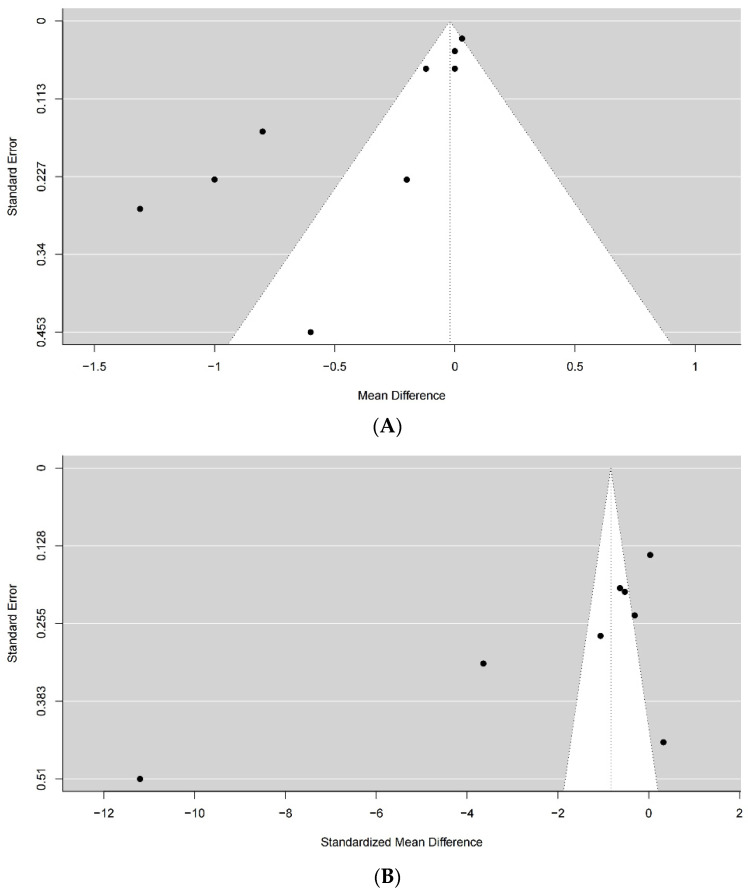

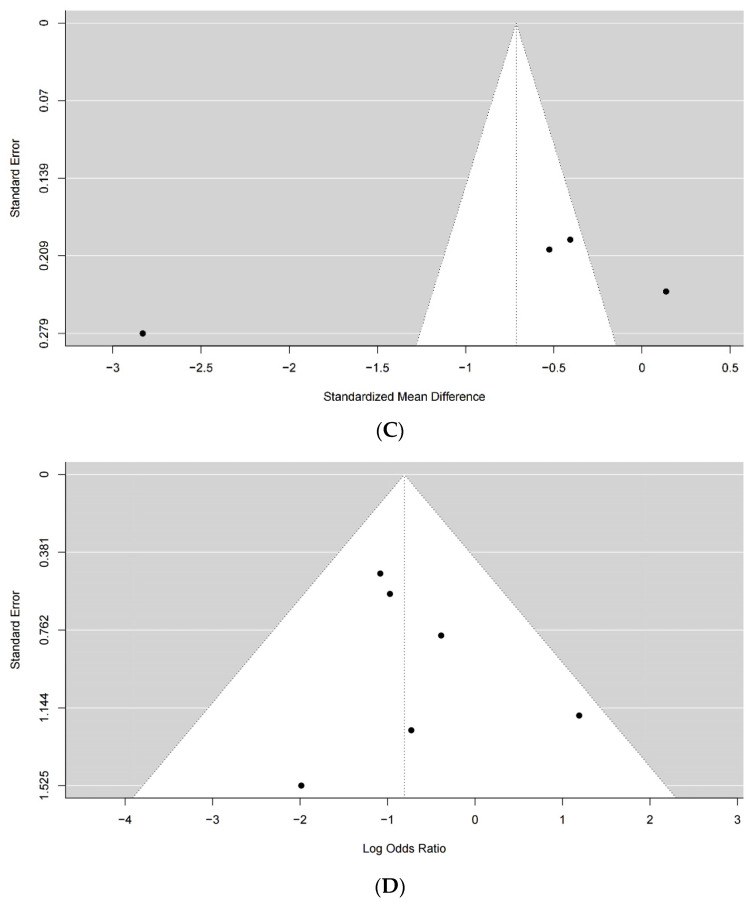
Funnel plots of the meta-analysis. Hemoglobin decline (**A**), volume of drainage (**B**), total blood loss (**C**), allogenic blood transfusion (**D**).

Funnel plots showed that there was no publication bias (Figure 6).

### 3.6. Complications

Complications, including superficial infection, deep infection, and venous thromboembolism (VTE), were investigated in five studies. In Kim et al.’s study [27], two superficial infections were reported in each of the Floseal and control groups. In Bae et al.’s study [22], VTE occurred in seven and nine cases in groups of thrombin-based hemostatic matrix and control, respectively. However, there was no case of deep infection that could be related to the use of a thrombin-based hemostatic matrix in any of the studies.

## 4. Discussion

This meta-analysis aimed to evaluate the effectiveness and safety of a thrombin-based hemostatic matrix in TKA based on up-to-date RCTs. The analysis focused on several key outcomes, including hemoglobin decline, the volume of drainage, total blood loss, and the risk of allogeneic transfusion. The results of the meta-analysis indicate that the use of a thrombin-based hemostatic matrix in TKA has a significant impact on reducing hemoglobin decline and the need for allogenic transfusion.

There has been a debate on the effectiveness of the application of topical hemostatic agents in TKA. Two previous meta-analyses have shown promising results regarding the effectiveness of thrombin-based hemostatic matrix in TKA. In 2014, Wang C. et al. reported that there was a significant advantage in hemoglobin decline and calculated total blood loss but no difference in postoperative drainage volume and rate of transfusion in TKA when using a thrombin-based hemostatic matrix [30]. In contrast, Fu X. et al. found that there was a significant difference in hemoglobin decline, total blood loss, drainage volume, and transfusion rate in the Floseal group compared to the control group [29]. Although there is no other previous meta-analysis on the use of Floseal in TKA, in two retrospective studies, Schwab PE demonstrated that there were no differences in hemoglobin and transfusion rate in patients who received minimal invasive TKA with or without aspirin [36,37]. Among the eight studies evaluated in this meta-analysis, RCTs by Yen SH et al. and Kim HJ et al. showed no advantage of Floseal in terms of hemoglobin level, transfusion rate, drainage volume, and total blood loss [27,35]. In contrast, studies by Bae KC et al. and Di Francesco A. et al. favored the use of Floseal in TKA [24,34]. Through this meta-analysis, we found advantages in using a thrombin-based hemostatic matrix in reducing hemoglobin decline and allogenic transfusion. With current meta-analyses, we added to the collective evidence in favor of the use of thrombin-based hemostatic matrix in TKA, along with other studies that support its use as a part of patient blood management.

In this meta-analysis, three studies [22,24,28] reported the effectiveness of a thrombin-based hemostatic matrix in reducing blood loss, while two other studies showed no significant effect. The conflicting results can be explained by different surgical and blood management protocols. In Yen et al.’s study, minimal invasive TKA that minimizes soft-tissue injury and subsequent bleeding was performed, and it can be related to a reduced difference between Floseal and control groups [35]. However, in other studies, conventional TKAs were performed, or TKA types were not described. Different blood drainage protocols also can affect the results of the study. Kim et al.’s study that placed a drain with low pressure for 24 h [27], and Yen et al.’s study that maintained a vacuum bag for 12 h with no full compression, followed by full compression until removal showed no difference between thrombin-based hemostatic matrix and control groups [35]. However, Di Francesco et al.’s study that placed a drain with high vacuum pressure for 24 h showed reduced blood drainage and transfusion rate in the Floseal group [24]. The use of tranexamic acid (TXA) also influences the postoperative bleeding and the study results. However, only two recent studies reported that the Floseal group did not use TXA [25,35], and it is not clear whether TXA was used perioperatively in the other six studies [22,24,26,27,28,34]. The amount of the Floseal used can also affect the results. However, the amount of Floseal was almost similar among studies (seven studies: 10 mL Floseal; one study: 5 ml Floseal), and therefore, its effect is likely to be minimal. Also, funding may become an issue that affects study results. However, among the three studies with funding [24,27,34], Kim et al.’s study showed no difference in hemoglobin drop between the thrombin-based hemostatic matrix and control groups [27]. On the contrary, among the five studies without funding [22,25,26,28,35], Bae et al.’s study [22] and Helito et al.’s study [25] reported reduced blood drainage and hemoglobin drop in the thrombin-based hemostatic matrix group compared to the control group. From these results, it is difficult to say that funding had an effect on the results of this study.

Comparing our findings with previous studies, our meta-analysis provides more recent and comprehensive evidence regarding the effectiveness of thrombin-based hemostatic matrix in TKA. Previous meta-analyses by Smith et al. and Li et al. also explored the outcomes of the use of a thrombin-based hemostatic matrix in TKA but included fewer RCTs and were published in 2018 and 2017, respectively [22,29,30]. Our study incorporates additional RCTs published since then, thereby strengthening the overall evidence base.

One of the reasons that the thrombin-based hemostatic matrix was effective in hemoglobin decrease and transfusion but not in drainage and total blood loss might be because the latter indices do not include hidden blood loss in the interstitial area. In contrast, hemoglobin decreases, and the risk of allogeneic blood transfusion represents perioperative blood loss from a more systemic point of view. Applying the thrombin-based hemostatic matrix in TKA might not show a significant difference in the amount of drainage or measured total blood loss, but it could be effective in occult bleeding [30].

Although our study demonstrates the potential benefits of the thrombin-based hemostatic matrix in TKA, certain limitations should be acknowledged. First, the included studies varied in terms of patient characteristics, surgical techniques, and outcome measures, which may introduce heterogeneity and affect the generalizability of the results. Second, the follow-up durations in the included studies were relatively short, limiting the assessment of long-term outcomes. Third, limited statistical significance was observed in a few studies included in the meta-analysis: hemoglobin reduction was significant in two studies, total weight loss in three studies, and transfusion rate in one study. Integrating the findings from individual studies in a meta-analysis, especially when there are only a few studies with statistically significant results, can lead to greater heterogeneity in the results and exacerbate the influence of publication bias. We used a random-effect model rather than a common-effect model when the I^2^ > 50%, indicating severe heterogeneity. Future studies with larger sample sizes and standardized protocols are needed to further validate the findings of this meta-analysis.

## 5. Conclusions

In conclusion, our meta-analysis suggests that thrombin-based hemostatic matrix is an effective hemostatic agent in TKA, leading to reduced hemoglobin decline, lower transfusion requirements, and improved postoperative outcomes. These findings provide valuable insights for orthopedic surgeons and enhance the existing evidence base. Further well-designed RCTs with longer follow-up periods are warranted to assess the long-term efficacy and safety of thrombin-based hemostatic matrix in TKA.

## Figures and Tables

**Table 1:** Demographic features of the included studies.

Author	Year	Number (F/C)	Age (Years) (F/C)	Male (F/C)	BMI (kg/m^2^) (F/C)	Antithrombotic Agent	Transfusion Criteria (g/dL)	Dosage (mL)
Kim HJ [27]	2012	97/99	72.7/70.1	N/S	N/S	Aspirin or warfarin	N/S	10
Helito CP [26]	2013	10/10	67.8/66.6	N/S	N/S	Enoxaparin	V/S change ^a^	10
Di Francesco A [24]	2013	51/42	67.9/70.2	24/17	26.0/26.2	Enoxaparin	Hb 8.5	10
Suarez JC [28]	2014	56/52	65.9/65.1	20/21	29.8/33.7	Enoxaparin	Hb 8.0	5
Bae KC [22]	2014	50/50	68.8/69.0	4/8	26.4/24.8	N/S	Hb 8.5	10
Velyvis JH [34]	2015	157/100	72.5/73.0	71/47	N/S	N/S	Hb 8 or 9 and associated symptoms ^b^	10 or 5
Helito CP [25]	2019	30/30	N/S	N/S	N/S	Enoxaparin	N/S	10
Yen SH [31]	2021	34/35	69.7/69.7	6/3	29.4/28.6	Enoxaparin	N/S	10

F/C, Floseal group/control group; BMI, body mass index; N/S, not stated; Hb, hemoglobin; V/S, vital sign. V/S change ^a^: heart rate >120 with mean arterial blood pressure < 80 mmHg or blood pressure < 100 mmHg (systolic) and 60 mmHg (diastolic), pulse oximetry < 90%, and tachypnea. Associated symptoms ^b^: weakness, dizziness, fainting, slow capillary refill, shortness of breath, or hypotension.

**Table 2:** Risk of bias of the included studies.

Author	Year	D1	D2	D3	D4	D5	Overall
Kim HJ [27]	2012	Low risk	Low risk	Low risk	Low risk	Low risk	Low risk
Helito CP [26]	2013	Low risk	Low risk	Low risk	Low risk	Low risk	Low risk
Di Francesco A [24]	2013	Some concerns	Low risk	Low risk	Some concerns	Low risk	Some concerns
Suarez JC [28]	2014	Low risk	Low risk	Low risk	Low risk	Some concerns	Some concerns
Bae KC [22]	2014	Some concerns	Low risk	Low risk	Low risk	Some concerns	Some concerns
Velyvis JH [34]	2015	Low risk	Low risk	Low risk	Low risk	Low risk	Low risk
Helito CP [25]	2019	Some concerns	Low risk	Low risk	Some concerns	Some concerns	Some concerns
Yen SH [35]	2021	Some concerns	Low risk	Low risk	Low risk	Low risk	Some concerns

D1: Randomization process; D2: deviations from intended interventions; D3: missing outcome data; D4: measurement of the outcome; D5: selection of the reported result.

## Data Availability

The data presented in this study are available on reasonable request from the corresponding author.
